# Influence of Hemianopic Visual Field Loss on Visual Motor Control

**DOI:** 10.1371/journal.pone.0056615

**Published:** 2013-02-15

**Authors:** Diederick C. Niehorster, Eli Peli, Andrew Haun, Li Li

**Affiliations:** 1 Department of Psychology, The University of Hong Kong, Hong Kong, Hong Kong Special Administrative Region, People's Republic of China; 2 Schepens Eye Research Institute, Massachusetts Eye and Ear Infirmary, Department of Ophthalmology, Harvard Medical School, Boston, Massachusetts, United States of America; CSIC-Univ Miguel Hernandez, Spain

## Abstract

**Background:**

Homonymous hemianopia (HH) is an anisotropic visual impairment characterized by the binocular inability to see one side of the visual field. Patients with HH often misperceive visual space. Here we investigated how HH affects visual motor control.

**Methods and Findings:**

Seven patients with complete HH and no neglect or cognitive decline and seven gender- and age-matched controls viewed displays in which a target moved randomly along the horizontal or the vertical axis. They used a joystick to control the target movement to keep it at the center of the screen. We found that the mean deviation of the target position from the center of the screen along the horizontal axis was biased toward the blind side for five out of seven HH patients. More importantly, while the normal vision controls showed more precise control and larger response amplitudes when the target moved along the horizontal rather than the vertical axis, the control performance of the HH patients was not different between these two target motion experimental conditions.

**Conclusions:**

Compared with normal vision controls, HH affected patients' control performance when the target moved horizontally (i.e., along the axis of their visual impairment) rather than vertically. We conclude that hemianopia affects the use of visual information for online control of a moving target specific to the axis of visual impairment. The implications of the findings for driving in hemianopic patients are discussed.

## Introduction

Homonymous hemianopia (HH) is an anisotropic visual impairment characterized by the binocular inability to see one side of the visual field. It is a common consequence of postchiasmic damage to the visual cortex due to cerebrovascular strokes on one side of the brain [1]. HH frequently affects patients' daily life, e.g., patients report bumping into objects or people [1] and have difficulties in driving a car [2].

### Visual perception of space in hemianopia

At the level of basic visual function, it has been shown that HH affects visual perception of space. A well-researched phenomenon is the hemianopic line bisection error. HH patients without neglect show a small bias (about 1°) toward their blind side when asked to bisect a line (e.g., [3], [4], [5], [6]). Note that this is a contralesional bias (i.e., ipsilateral to the visual field defect), whereas neglect patients normally show a larger ipsilesional bias when performing the line bisection task [4].

Closely related to the line bisection error is the shift of the perceived straight ahead in HH patients. Ferber & Karnath [7] asked patients to move a light, initially randomly positioned in a dark room, to their perceived straight ahead. They found that HH patients on average showed an 8° shift of their perceived straight ahead toward their blind side (i.e., contralesional), whereas neglect patients showed a 5° shift away from their neglect side (i.e., ipsilesional). Together with the hemianopic line bisection error, this bias of the perceived straight-ahead observed in HH patients suggests that homonymous field defects are associated with visual misperception of space [8]. Such misperception arises possibly due to the tendency of HH patients to compensate for their visual deficit by maintaining a fixation position somewhat into their blind visual field [9], [10] or making more exploratory eye movements into their blind field [11].

### Hemianopia and driving

In many countries and over half of the states in the U.S., HH patients are not allowed to drive due to legal restrictions regarding the minimum size of the visual field [12], [13], [14], [15], [16]. However, in some states in the U.S., and in countries such as the Netherlands, Belgium, the UK and Canada, HH patients can be issued a driving license after successful completion of an on-road test [14], [17].

Depending on the specific driving situation tested and on the patient selection criteria, previous studies have found that the driving ability of HH patients varies widely [18], [19], [20], [21]. The percentage of HH patients judged unfit to drive varied between 27% [20], [21] and 86% [18]. While some work has shown that hazard detection can be an issue [22], [23], the most frequent reasons for failing the driving tests are problems related to unstable steering revealed by lane position variability [18], [20], [21]. Furthermore, several studies employing driving simulators (e.g., [2], [24], [25]) have reported that compared with normal vision controls, HH patients show not only a more variable lane position but also a tendency to increase the space between the car and the lane edge on their blind side.

### Closed-loop visual motor control

Lane-keeping is a common aspect of real world driving. Both external factors (such as crosswinds, bumps in the road surface, road curves and tire imbalance) as well as factors internal to the driver (such as the driver's driving skills and attentional state) continuously affect the vehicle's position in the lane. To keep the vehicle in the center of the lane requires the driver to constantly use visual feedback to quickly and effectively minimize the vehicle's lane position error, which is a closed-loop visual motor control task [26], [27]. The ability to use available visual cues to minimize lane position errors to maintain a stable lane position is important for safe driving.

Previous studies have shown that lane-keeping control can be successfully approximated by the control performance on a display that simulated an observer driving a vehicle down the lane under pseudo-random crosswind perturbations [28], [29], [30], [31]. It has also been shown that human operators can perform such a closed-loop visual motor control task under various controller dynamics [32], [33], [34]. Based on these findings, Li et al. have developed a simple closed-loop visual motor control task that involves controlling a randomly moving visual target on the screen to evaluate how the visual system uses different sources of visual information for the control of target motion [35], [36], and how the recruitment of new visual information for visual motor control is affected by controller dynamics [37].

### The current study

Although previous studies have consistently found that HH patients show increased lane position variability during driving [2], [18], [19], [20], [21], [25], a detailed analysis of their visual motor control abilities has not been performed. In the current study, we used a simple closed-loop visual motor control task similar to that developed by Li et al. [35], [36], [37] to evaluate the visual motor control abilities of HH patients. The goal was to compare the control performance of HH patients to that of a group of age-matched normally sighted participants to determine the extent to which the visual impairment in HH patients affects their ability to effectively use visual information for online control of a moving target.

**Figure 1:**
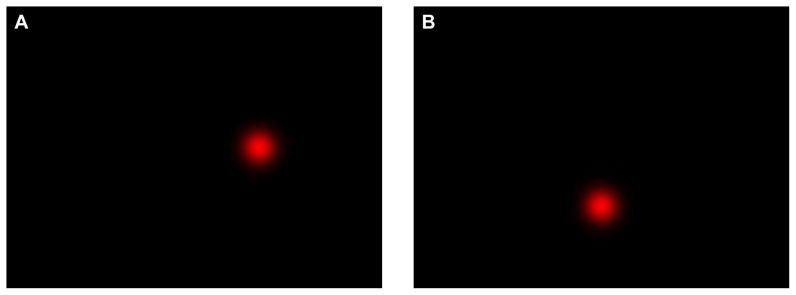
Schematic illustrations of the displays used in the study. (a) The target moves along the horizontal axis of the screen and displays a rightward error from the center of the screen, and (b) the target moves along the vertical axis of the screen and displays a downward error from the center of the screen.

Specifically, the display showed a target moving along either the horizontal or the vertical axis of the screen while undergoing pseudo-random perturbations (Figure 1). Both a HH patient group and a normal vision control group were asked to use a joystick to keep the target as close to the center of the screen as possible. While similar closed-loop visual motor control tasks have been used to characterize motor impairment due to neurological disorders [38], [39], [40] or alcohol intake [41], [42], [43], the current study is the first investigation to examine the changes in visual motor control due to visual impairments.

The motivation of the study design is given as follows: the aforementioned line bisection error and the shift in the perceived straight-ahead in HH patients [7], [8] show that HH affects visual perception along the horizontal but not the vertical axis. If such anisotropic distortions of the visual input affect visual motor control, we expect that compared with normal vision controls, the control performance of HH patients should be affected when the target moves horizontally but not when it moves vertically. However, if HH or the associated brain damage has a general impact on visual motor control, the control performance should degrade regardless of whether the target moves horizontally or vertically.

Note that although it has been reported that in addition to the visual impairment, right HH due to damage to the left hemisphere of the brain is frequently accompanied by reading and language deficits (e.g., [44], [45], [46]), and left HH due to the damage to the right hemisphere is frequently associated with neglect and topographical disorientation (e.g., [47], [48], [49]), the within-subject design of the current study allows us to isolate the effect of the visual impairment on visual motor control in HH from that of other non-visual impairments. Presumably, non-visual impairments, which might be different for left HH and right HH patients, would similarly affect the control performance along both axes. The visual impairment on the other hand would be expected to have a larger effect on the patients' control performance along the axis of their visual impairment, i.e., when the target moves horizontally on the display.

## Methods

### Participants

**Figure 2:**
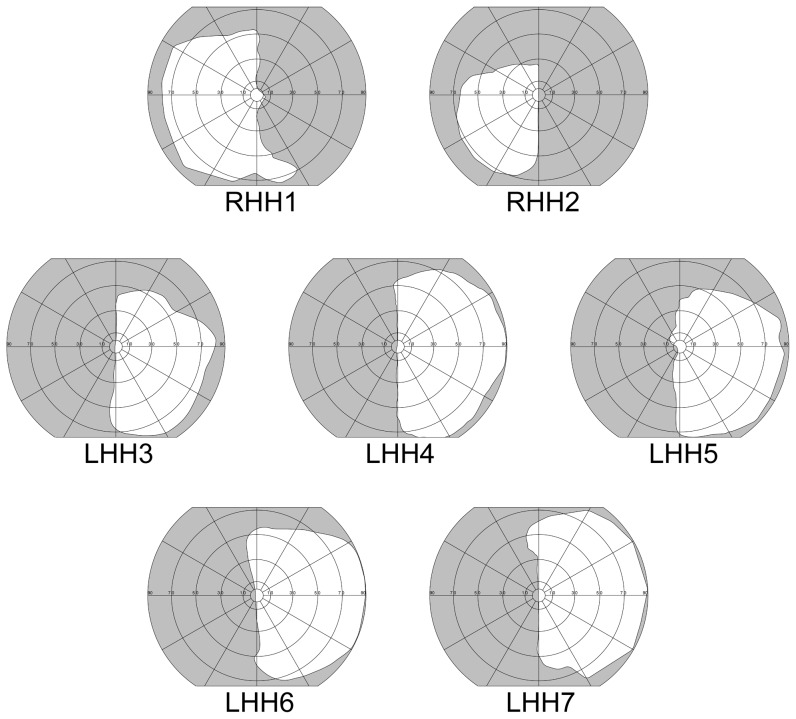
HH patients' binocular visual fields. The binocular visual fields of the seven HH patients are indicated by the white areas. Patients reported no vision in the gray-shaded areas, as measured with a V4e target for Goldmann kinetic perimetry.

Seven HH patients were recruited from a patient database at Schepens Eye Research Institute. These patients had complete hemianopia (see criteria in [50]), no spatial neglect as tested with the Bells test [51] and the Schenkenberg Line Bisection test [52], no significant cognitive impairment as tested with the MiniMental State Examination test (MMSE ≥24 [53]), and corrected visual acuity of 20/40 OU or better. The HH of all patients was stable as the onset of their HH occurred at least three years prior to their participation in the experiment (see [54]). All but two patients (RHH1 and RHH2 in Figure 2) had left HH. All patients performed the task with their dominant hand. One patient (LHH7) had hemiparesis but this did not affect his ability to do the control task as he used his unaffected and dominant hand to control the joystick. Table 1 summarizes the demographics of the seven HH patients and Figure 2 displays graphs of their visual field deficits as assessed with a Goldmann V4e target.

**Table 1:** Demographics of the HH patients.

Patients	age	gender	side of HH	HH cause	years since onset
RHH1	35	F	R	stroke	4
RHH2	76	M	R	stroke	10
LHH3	57	M	L	stroke	3
LHH4	52	F	L	stroke	9
LHH5	33	M	L	tumor removal	18
LHH6	81	M	L	stroke	10
LHH7	59	M	L	stroke	4

A comparison group of seven participants with normal or corrected-to-normal vision was recruited. The control participants were each matched in gender and age (within 5 years, *t*(12) = 0.12, *p* = 0.91) to one of the seven HH patients. None of the patients or controls had previous experience with the control task. The study was conducted in accordance with the tenets of the Declaration of Helsinki and was approved by Institutional Review Boards at The University of Hong Kong and Schepens Eye Research Institute. Written informed consent was obtained from all participants.

### Visual stimuli and experimental setup

A red round Gaussian target (σ: 0.6°, peak luminance: 9.4 cd/m^2^) was displayed on a 21″ CRT monitor (1280×960 pixels) on a uniform black background (0.07 cd/m^2^) at a 100 Hz refresh rate (Figure 1). Participants were seated in a darkened room at a viewing distance of approximately 50 cm where the display subtended a visual angle of 41° (H)×31° (V).

Two target motion conditions were tested: in (1) the *horizontal* condition, the target's horizontal position on the screen was perturbed, while in (2) the *vertical* condition, the target's vertical position was perturbed. The input position perturbation 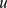 consisted of the sum of seven harmonically unrelated sinusoids and is given as a function of time 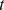 by
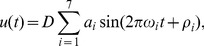
(1)where 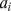 and 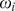 respectively represent the amplitude and frequency of the 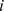 ^th^ sine component (Table 2), and 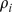 is a random phase offset drawn each trial from the range 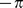 to 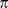. Disturbance gain 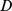 was set to a value of 2.3°/s, which led to an average uncorrected perturbation speed of 6.6°/s (peak: 24°/s). This sum-of-sinusoids perturbation series made the target's motion appear random and allowed for a frequency-based analysis of the controller's response.

**Table 2:** Input position perturbation signal.

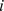	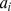	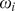 (Hz)
1	2	0.1
2	2	0.14
3	2	0.24
4	2	0.41
5	0.2	0.74
6	0.2	1.28
7	0.2	2.19

Amplitudes (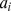) and frequencies (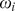) of the seven harmonically independent sinusoids for the input position perturbation 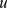.

The participants were asked to use a joystick (Flybox, B&G Systems, Palo Alto, CA) to control the moving target and keep it centered on the screen. Participants moved the joystick left-to-right to control the target's horizontal movement in the horizontal condition, or fore-to-aft to control the target's vertical movement in the vertical condition. We used velocity controller dynamics in which the joystick displacement, sampled at 100 Hz, was proportional to the target's velocity on the screen. This controller dynamic is similar to that of the steering wheel of a vehicle. The end-to-end system feedback delay was two frames (20 ms).

### Procedure

**Figure 3:**
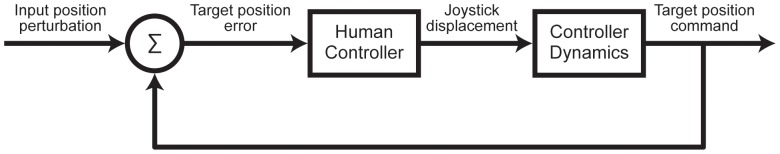
Block diagram depicting the closed loop active control task.

At the beginning of each 95 second trial, the target appeared at the center of the screen and began moving when participants pulled the trigger of the joystick. Initially, the target moved according to the sum-of-sinusoids perturbation, but as participants moved the joystick to keep the target at the center of the screen, the target's position was affected by the sum of the controller's target position command and the input perturbation (see Figure 3). Participants were asked to track the target's movement on the screen and to make smooth control adjustments to keep the target as close to the center of the screen as possible.

Participants performed the horizontal and vertical target motion conditions in separate blocks. The testing order of these conditions was counterbalanced between participants. Each block started with practice trials to familiarize participants with the task and the joystick controller dynamics. The practice continued until the control performance plateaued, which required 4–8 trials for both the HH patients and the normal vision controls. Participants then completed eight experiment trials. Participants started each trial at their own pace and were given ample break time between blocks and between the training and data collection parts of each block. Participants completed the experiment in a single session, lasting 1.5 to 2 hours.

### Data analysis

We calculated the mean deviation of the target position from the center of the screen (i.e., the mean target position error) for each trial, which indicates the participant's perceived center of the screen. We furthermore computed several metrics to evaluate the control performance. First, the total control error was measured as the root mean square (RMS) of the time series of the target position relative to the mean target position during the trial. The RMS error indicates the precision with which participants were able to maintain the target at their perceived center of the screen.

Second, to evaluate the control response specific to the different input perturbation frequencies, we performed a frequency-response (Bode) analysis to obtain the response gain and phase lag at each perturbation frequency. Specifically, we performed Fourier analysis of the time series of the target position error and the joystick displacement in each trial. The response gain and phase lag at each perturbation frequency were then computed by taking the ratio of the Fourier coefficients of the target position error and joystick displacement data at the input perturbation frequencies. For all analyses, the first 5 s of data in each 95 s trial were omitted to ensure that only the steady state control response was analyzed.

## Results

### Mean target position error

**Figure 4:**
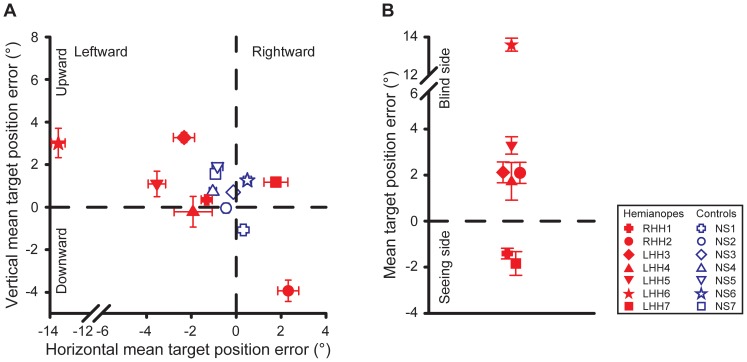
Mean target position error. (a) Mean target position error for the horizontal and the vertical target motion conditions for each participant in the patient and the control groups. Errors were similar for the patient and the control groups. (b) Mean target position error for the horizontal target motion condition recoded such that positive values correspond to a bias in the perceived center of the screen toward the patient's blind side, and negative values toward the seeing side. Five out of seven patients showed a bias toward their blind side. Error bars in both panels indicate SEs across eight trials.

The mean target position error averaged across eight trials for the horizontal target motion condition is plotted against that for the vertical condition for each participant in both the patient and the control groups in Figure 4a. For both the horizontal and vertical target motion conditions, there was no significant difference in the mean target position error between the HH patient group and the control group (*t*(12) = −1.14, *p* = 0.28 and *t*(12) = −0.04, *p* = 0.97, respectively).

As the mean target position error measured the perceived center of the screen, for each HH patient, we then recoded the mean target position error from the horizontal target motion condition into a bias in the perceived center of the screen toward the patient's blind or seeing side (Figure 4b). The findings showed that the mean bias (mean±SE: 3.1°±2.3°) in the patients' perceived center of the screen was toward their visual field loss. However, a one sample t-test did not find this bias significantly different from zero (*t*(6) = 1.53, *p* = 0.18), possibly due to the large variation in the individual data. Nevertheless, for five out of the seven HH patients tested, the bias was toward their visual field loss. The direction and the mean magnitude of the biases observed in these five patients were consistent with the previously reported biases toward the visual field loss in the line bisection tasks and the perceived straight-ahead judgments in HH patients (e.g., [12], [16]).

One HH patient (LHH6) showed an exceptionally large bias (13.6°) toward his blind side. This patient remarked that he felt that the screen extended into his blind side much further than it actually did. Because of this, he might have perceived the center of the screen to be close to the edge of the screen at his blind side.

### Overall control performance error

**Figure 5:**
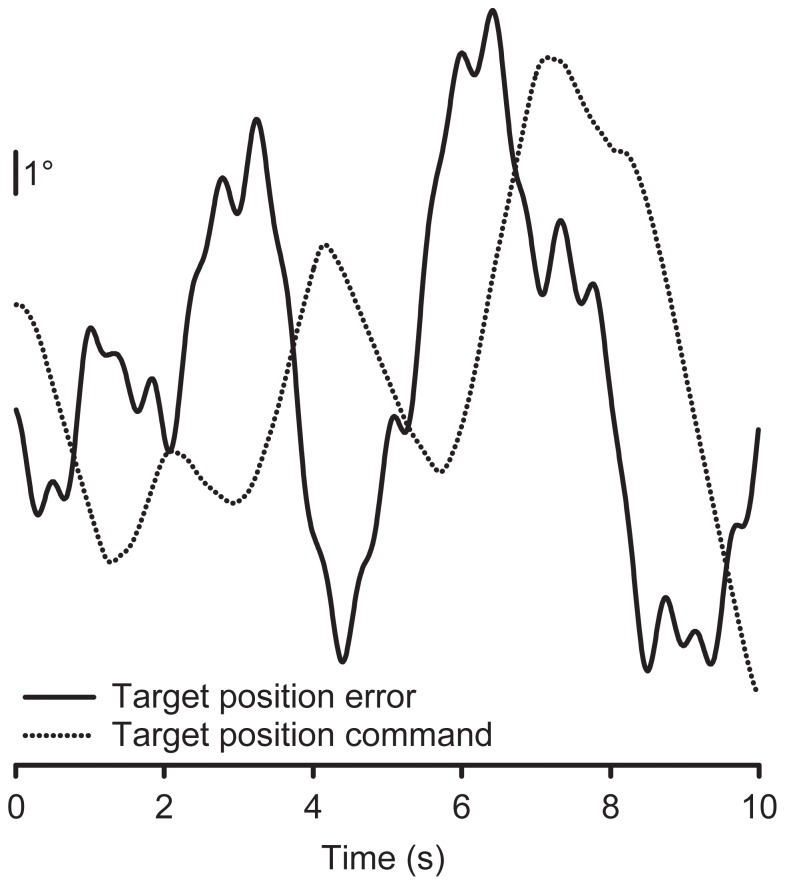
Raw performance data of a HH patient for the horizontal target motion condition. The solid line depicts the input target position error and the dotted line depicts the output target position command, which is a smoothed out and delayed version of the input.

Figure 5 plots the input target position error and the output target position command (see Figure 3) generated by a HH patient for a representative section of data for the horizontal target motion condition. As can be seen from the plot, the patient's control response was a scaled and delayed version of the input target position error, with some smoothing out of the response at the highest frequencies. The data for both target motion conditions and for both the patient and the control groups showed a similar relationship between the input position error and the output control response.

**Figure 6:**
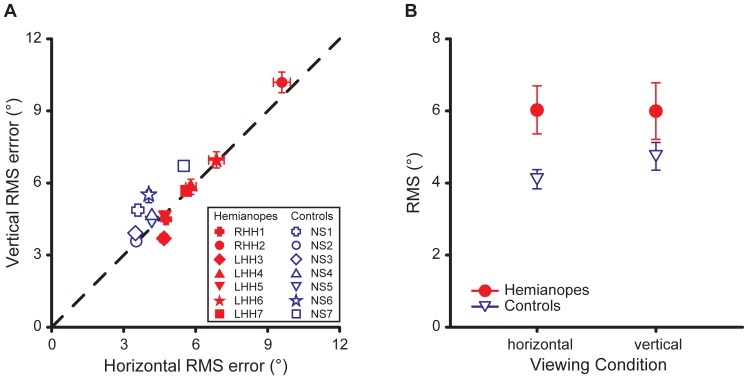
RMS target position error. (a) Mean RMS target position error for the horizontal target motion condition against that for the vertical condition for each participant in the two participant groups. Error bars indicate SEs across eight trials. (b) Mean RMS target position error averaged across seven participants for the patient and control groups for the horizontal and the vertical target motion conditions. Error bars are SEs across seven participants. While mean RMS target position error was lower for the horizontal than the vertical target motion condition for the control group, there was no difference in mean RMS error between the two target motion conditions for the patient group.

The mean RMS target position error averaged across eight trials for the horizontal target motion condition is plotted against that for the vertical target motion condition for each participant in Figure 6a. The 45° diagonal line (a unity slope) indicates equal RMS error in the horizontal and vertical target motion conditions, while data that lay above the diagonal correspond to larger RMS error in the vertical than in the horizontal target motion condition, and data that lay below the diagonal correspond to larger RMS error in the horizontal than in the vertical target motion condition. Wilcoxon Signed Rank tests revealed that while the RMS error of the normal vision controls was smaller in the horizontal than in the vertical condition (*z* = 2.37, *p* = 0.018), the control performance of the HH patients was similar in both target motion conditions (*z* = 0.0, *p* = 1.0). Note that after the experiment during debriefing, both the HH patients and normal vision controls reported that they found controlling horizontal target motion easier than controlling vertical target motion. The perceived ease of controlling horizontal target motion could be due to the fact that left-to-right joystick control involves less movements of the arm compared with the front-to-aft joystick control.

To further compare the control performance of the patients with that of the normal vision controls, the mean RMS error, averaged over seven participants for each group, is plotted against target motion condition in Figure 6b. A 2 (target motion condition)×2 (participant group) mixed design ANOVA revealed that the main effect of target motion condition and the interaction effect of target motion condition and the participant group were both significant (*F*(1,12) = 7.06, *p* = 0.021 and *F*(1,12) = 8.71, *p* = 0.012, respectively). Although the main effect of participant group was marginally significant (*F*(1,12) = 4.05, *p* = 0.07), Newman-Keuls tests did not reveal significant differences between the control gains of the two participant groups for either the vertical (*p* = 0.14) or the horizontal (*p* = 0.13) target motion conditions. Consistent with the slope data mentioned above, Newman-Keuls tests showed that while the control group produced more precise control in the horizontal than the vertical target motion condition (4.11° vs. 4.74°, *p* = 0.002), the HH patient group showed similar RMS errors for the two target motion conditions (6.03° vs. 6.00°, *p* = 0.84).

### Frequency-specific performance

**Figure 7:**
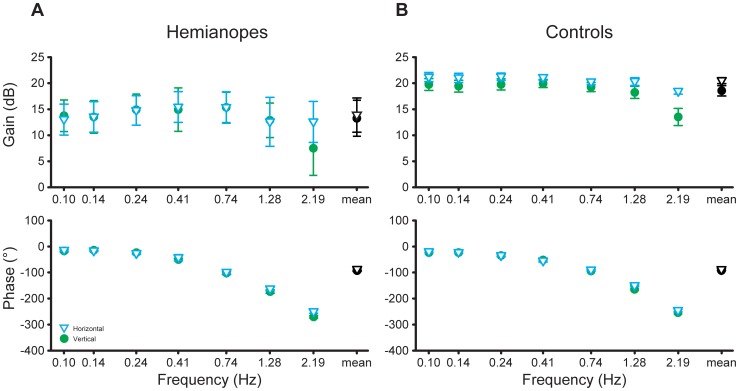
Frequency-response (Bode) plots of control performance. The top panels present mean gain and the bottom panels mean phase lag, averaged over seven participants, for (a) the HH patient group and (b) the normal vision control group. The rightmost points in each panel indicate mean gain (upper panels) or phase lag (lower panels), averaged across the seven frequencies. Error bars are SEs across seven participants. While mean response gain was higher for the horizontal than the vertical target motion condition for the control group, there was no difference in mean response gain between the two target motion conditions for the patient group. Mean phase lag was lower for the horizontal than the vertical target motion condition for both participant groups.

A frequency-specific analysis of the control performance allows us to look at changes in response gain and phase lag at each input perturbation frequency. Figure 7 plots the response gains and phases as a function of input perturbation frequency for the HH and the control groups for both target motion conditions. The decreasing response gain and the steady phase roll-off at high frequencies are consistent with the low-pass gain control with a time delay typically observed in previous active control studies [35], [36], [55].

To examine the difference in the response gain between the two participant groups for each target motion condition, we averaged the response gain over all seven perturbation frequencies (Figure 7, the rightmost data points in the upper panels). A 2 (target motion condition)×2 (participant group) mixed design ANOVA revealed that the interaction effect of target motion condition and the participant group was significant (*F*(1,12) = 6.06, *p* = 0.030), the main effect of target motion condition was marginally significant (*F*(1,12) = 4.17, *p* = 0.064) and the main effect of participant group was not significant (*F*(1,12) = 2.85, *p* = 0.12). Newman-Keuls tests showed that while for the control group, the response gain was higher in the horizontal than the vertical target motion condition (20.4 dB vs. 18.4 dB, *p* = 0.008), for the patient group, the response gain was similar for the two target motion conditions (13.5 dB vs. 13.7 dB, *p* = 0.77).

**Figure 8:**
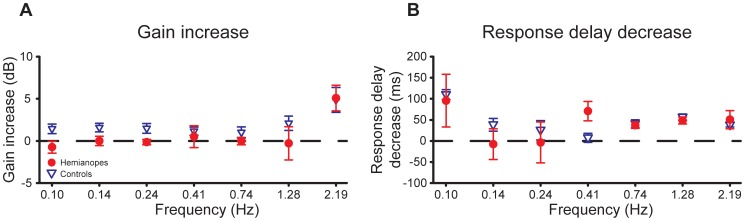
Gain and response delay differences between conditions. (a) Mean gain increase from the vertical to the horizontal target motion condition. Across the six lower frequencies, the control group showed a larger increase in gain from the vertical to the horizontal condition than did the patient group. (b) Mean response delay of the horizontal condition relative to that of the vertical condition against the seven perturbation frequencies. Mean relative response delay was similar for both participant groups at all frequencies. Error bars are SEs across seven participants.

To examine how the difference in the response gain between the two target motion conditions changes with the input perturbation frequency, for each participant group, we calculated the change in gain by subtracting the control gain of the vertical condition from that of the horizontal condition at each perturbation frequency (Figure 8, left panel). Both groups showed a much larger increase in gain from the vertical to the horizontal condition at the highest perturbation frequency. A 6 (frequency)×2 (participant group) mixed design ANOVA on gain increases at the six lower frequencies revealed that the main effect of participant group was significant (*F*(1,12) = 4.92, *p* = 0.047), and the main effect of frequency and the interaction effect of frequency and participant group were not (*F*(5,60) = 0.16, *p* = 0.98 and *F*(5,60) = 0.36, *p* = 0.87, respectively). Across the six lower frequencies, the control group showed a larger increase in gain from the vertical to the horizontal target motion condition than did the patient group.

To examine the difference in the response phase lag between the two participant groups for each target motion condition, we averaged the phase lag over all seven frequencies (Figure 7, the rightmost data points in the lower panels). A 2 (target motion condition)×2 (participant group) mixed design ANOVA revealed that the main effect of target motion condition was significant (*F*(1,12) = 5.70, *p* = 0.034), and that the main effect of participant group and their interaction effect were not significant (*F*(1,12) = 0.0018, *p* = 0.97 and *F*(1,12) = 0.16, *p* = 0.70, respectively). The mean phase lag for the vertical target motion condition (93.5°) was larger than that for the horizontal target motion condition (88.9°). This indicates a faster control response in the horizontal than in the vertical target motion condition across both participant groups, which is consistent with the participants' report that front-to-aft joystick control in the vertical target motion condition is physically more difficult to perform than the left-to-right joystick control in the horizontal target motion condition.

To examine whether the reduction in phase lag for the horizontal target motion condition corresponded to a systematic reduction in time delay of the control response, we calculated the relative response delay between the two target motion conditions. That is, we first converted phase lag to response delay by dividing each phase by the corresponding frequency multiplied by 360° and then subtracted the response delay for the horizontal condition from that for the vertical target motion condition (Figure 8, the right panel). A 7 (frequency)×2 (participant group) mixed design ANOVA revealed no significant effects (*p*>0.145), indicating that the relative response delay was similar for both participant groups at all frequencies.

## Discussion

We investigated how hemianopic visual field loss affects visual motor control by comparing the control performance of a HH patient group to that of a normal vision control group. Both groups were tested in two target motion conditions in which a target moved either horizontally or vertically on a computer screen. While the control group on average showed a 13% reduction in RMS target position error and a 25% increase in response gain for the horizontal compared with the vertical target motion condition, the HH patients showed similar performance in both target motion conditions. Nevertheless, both groups showed on average 23 ms faster control responses for the horizontal than the vertical target motion condition. In summary, these results indicate that while HH affects the precision and the amplitude of the control response specific to the axis of the visual impairment, it does not have a significant effect on response time.

Patients had to make eye movements to follow the target's movement on the screen to perform the task in the horizontal target motion condition, as otherwise the target would have disappeared into their blind side. However, we did not monitor eye movements and thus cannot exclude the possibility that the patients occasionally lost the target in their blind visual field. No patient however reported having difficulties in following the target, and it is hard to think of a pattern of eye movements that would lead to an impairment of gain but not response time for the control of horizontal target motion. Thus, the reported interaction between participant group and target motion condition is not likely due to patients losing sight of the target. Instead, we propose that the misperception of visual space along the horizontal axis in HH patients [4], [7], [8], [56] affected their use of visual information for online control of horizontal target motion, leading to higher RMS control error and lower control gains.

Given the similar requirements of closed-loop visual motor control for lane-keeping and the target motion control task used in the current study, the finding that HH leads to impaired visual motor control specific to the axis of the visual impairment has implications for driving in HH patients. Indeed, the results of the currents study correspond to those of hemianopic driving studies. To illustrate, the larger RMS target position error for the horizontal target motion condition observed in HH patients compared with normal vision controls agrees with the increased lane position variability in HH patients from previous driving studies [2], [18], [20], [21], and the smaller response gain for the horizontal target motion condition in HH patients is also consistent with the less efficient steering to correct lane position reported by Bowers et al. [2].

As the current study is the first investigation that showed impaired visual motor control due to partial visual field loss, future research should address whether rehabilitation techniques that allow HH patients to compensate for their visual deficits [17], [57], [58], [59] such as appropriate scanning training [60], [61], [62] and optical aids [63], [64] may also help restore their visual motor control abilities. As many activities of daily life involve closed-loop visual motor control, such a research program might ultimately increase the mobility of HH patients. Note that as high power optical aids or changes in the use of eye movement strategy can introduce spatial distortions of their own and may thereby further impair steering performance, these rehabilitation methods need to be carefully studied in the context of driving.
